# Asian Dust Particles Induce Macrophage Inflammatory Responses via Mitogen-Activated Protein Kinase Activation and Reactive Oxygen Species Production

**DOI:** 10.1155/2014/856154

**Published:** 2014-05-29

**Authors:** Kazuma Higashisaka, Maho Fujimura, Mayu Taira, Tokuyuki Yoshida, Shin-ichi Tsunoda, Takashi Baba, Nobuyasu Yamaguchi, Hiromi Nabeshi, Tomoaki Yoshikawa, Masao Nasu, Yasuo Yoshioka, Yasuo Tsutsumi

**Affiliations:** ^1^Laboratory of Toxicology and Safety Science, Graduate School of Pharmaceutical Sciences, Osaka University, 1-6 Yamadaoka, Suita, Osaka 565-0871, Japan; ^2^Laboratory of Biopharmaceutical Research, National Institute of Biomedical Innovation, 7-6-8 Saito-Asagi, Ibaraki, Osaka 567-0085, Japan; ^3^The Center for Advanced Medical Engineering and Informatics, Osaka University, 1-6 Yamadaoka, Suita, Osaka 565-0871, Japan; ^4^Laboratory of Environmental Science and Microbiology, Graduate School of Pharmaceutical Sciences, Osaka University, 1-6 Yamadaoka, Suita, Osaka 565-0871, Japan; ^5^Division of Foods, National Institute of Health Sciences, 1-18-1 Kamiyoga, Setagaya-ku, Tokyo 158-8501, Japan

## Abstract

Asian dust is a springtime meteorological phenomenon that originates in the deserts of China and Mongolia. The dust is carried by prevailing winds across East Asia where it causes serious health problems. Most of the information available on the impact of Asian dust on human health is based on epidemiological investigations, so from a biological standpoint little is known of its effects. To clarify the effects of Asian dust on human health, it is essential to assess inflammatory responses to the dust and to evaluate the involvement of these responses in the pathogenesis or aggravation of disease. Here, we investigated the induction of inflammatory responses by Asian dust particles in macrophages. Treatment with Asian dust particles induced greater production of inflammatory cytokines interleukin-6 and tumor necrosis factor-**α** (TNF-**α**) compared with treatment with soil dust. Furthermore, a soil dust sample containing only particles ≤10 **μ**m in diameter provoked a greater inflammatory response than soil dust samples containing particles >10 **μ**m. In addition, Asian dust particles-induced TNF-**α** production was dependent on endocytosis, the production of reactive oxygen species, and the activation of nuclear factor-**κ**B and mitogen-activated protein kinases. Together, these results suggest that Asian dust particles induce inflammatory disease through the activation of macrophages.

## 1. Introduction

Asian dust (also yellow sand) is a dominant springtime aerosol in East Asia. The dust originates from the deserts of East Asia, such as the Takla Makan and Gobi, and is spread by prevailing winds not only locally to China and Mongolia but also throughout East Asia to countries such as Korea, Taiwan, and Japan. A similar phenomenon originating from the Sahara has also been observed in Europe and the United States [1, 2], raising concerns throughout the world about the impacts of aeolian dust [3, 4].

Asian dust not only is responsible for financial loss through crop damage, tree collapse, and flight cancellations due to low visibility, but also it poses a major threat to human health. Recent reports have shown that, in addition to harmful chemicals, Asian dust particles contain lipopolysaccharides and *β*-glucan, which are components of the bacterial cell membrane and fungal cell wall, respectively, and that bacteria can adhere to the outer surface of the dust particles [5, 6]. Indeed, morbidity rates for cardiovascular and respiratory diseases in Central East Asian countries increase during Asian dust storms [7–9]; in Korea, Asian dust storms are correlated with a 2.2% increase in the rate of patients over the age of 65 presenting with respiratory symptoms [10]. The association between Asian dust levels and the pathogenesis of childhood asthma has been clearly demonstrated; Asian dust likely triggers the onset of this and other inflammatory conditions through the induction of excessive antigen-specific or nonspecific inflammatory responses [11].

Although most of the information regarding Asian dust-related health problems is based on epidemiological investigations, some experimental studies have demonstrated the effects of Asian dust on allergic respiratory diseases* in vivo* [12, 13]. For example, Asian dust particles are reported to enhance both ovalbumin-induced eosinophil recruitment in the alveoli and airway submucosa in mice [14] and nasal allergic reactions in guinea pigs [15]. However, detailed information on the mechanisms of these inflammatory responses remains limited. To further our knowledge on the mechanisms through which Asian dust affects human health, it is essential to evaluate the interplay among the physical characteristics and the biological responses it provokes.

It is generally accepted that, like bacteria and viruses, particulate matter such as Asian dust particles is eliminated from the human body by phagocytes such as macrophages [16, 17]. Macrophages ingesting exogenous materials produce interleukin-6 (IL-6) and tumor necrosis factor-*α* (TNF-*α*) and, through the activation of signal pathways such as the mitogen-activated protein kinase (MAPK) pathways, expedite the elimination of particulate matter by inducing inflammatory responses; however, excessive or chronic macrophage activation results in inflammatory diseases such as bronchitis or pneumonia [18]. It is therefore important to assess the biological responses of macrophages to Asian dust particles, in particular the inflammatory responses. In the present study, we collected samples of suspended Asian dust particles from Beijing, China, and soil dust from Loess Plateau, China, which is a source of Asian dust, and evaluated their effects on the macrophage inflammatory response.

## 2. Materials and Methods

### 2.1. Reagents

Samples of suspended Asian dust particles which fell on the top of the building of the Beijing inner city were collected each day for three successive days from March 24, 2010 (defined as ADP1), and once on March 20, 2010 (ADP2). Samples of soil dust were collected on June 30, 2009, from three locations at Loess Plateau, China, as follows: site 1: lat 35° 35′ 450′′N, long 109° 10′ 074′′E (SDP1); site 2: lat 35° 42′ 324′′N, long 109° 25′ 390′′E (SDP2); and site 3: lat 35° 42′ 279′′N, long 109° 25′ 450′′E (SDP3). These soil dust particles were the same samples previously used by Yamaguchi et al. in [6]. In addition, sample SDP2 was partitioned by using a soundwave vibrating screen (Tsutsui Rikagaku Kikai Co., Ltd., Tokyo), and particles ≤10 *μ*m in diameter were collected (SDP4). Lipopolysaccharide (LPS), butylated hydroxyanisole (BHA), broad-spectrum ROS scavenger, and diphenyleneiodonium chloride (DPI), a specific inhibitor of nicotinamide adenine dinucleotide phosphate (NADPH) oxidase, were purchased from Sigma-Aldrich (St. Louis, MO). U0126, an extracellular signal-regulated kinase (ERK) inhibitor, and SP600125, a c-Jun N-terminal kinase (JNK) inhibitor, were obtained from Merck (Darmstadt, Germany). SN50, a nuclear factor-*κ*B (NF-*κ*B) inhibitor, was purchased from Calbiochem (La Jolla, CA).

### 2.2. Cells

RAW264.7 cells (mouse monocyte/macrophage cell line) were obtained from the American Type Culture Collection (Manassas, VA) and cultured at 37°C in Dulbecco's modified Eagle's medium (DMEM; Wako Pure Chemical Industries, Osaka, Japan) supplemented with 10% fetal bovine serum and antibiotics.

### 2.3. Scanning Electron Microscopy Analysis

The Asian dust particles and soil dust samples were adjusted to 0.25 mg/mL with deionized water. Aliquots (50 *μ*L) of each sample were then dried on a hot plate (Cimarec, Barnstead Thermolyne, Dubuque, IA) and observed under a scanning electron microscope (Jeol, Ltd., Tokyo, Japan).

### 2.4. Cytotoxicity and Cytokine Production Assays

RAW264.7 cells (1.5 × 10^4^ cells/well) were seeded in 96-well plates (Nunc, Rochester, NY), cultured at 37°C for 24 h, and then treated with 6.25, 25, or 100 *μ*g/mL of a suspension of Asian dust particles or soil dust or DMEM (negative control) or 1.5 *μ*g/mL of LPS at 37°C for 24 h. The cytotoxicity of Asian dust was assessed by methylene blue assay. The cells were fixed with glutaraldehyde and stained with 0.05% methylene blue for 15 min. After washing with water, the methylene blue in the wells was eluted using 200 *μ*L of 0.33 N HCl for each of the wells and absorbance was measured at 655 nm (subwave length, 415 nm). Cell viability was calculated as the ratio of absorbance in the treated cultures compared to the control untreated cultures. Enzyme-linked immunosorbent assay (ELISA) kits were used in accordance with the manufacturer's instructions to determine the levels of IL-6 (BD Pharmingen, San Diego, CA) and TNF-*α* (eBioscience, San Diego, CA) in the culture supernatants.

### 2.5. Inhibition Assay

RAW264.7 cells (1.5 × 10^4^ cells/well) were seeded in 96-well plates (Nunc), cultured at 37°C for 24 h, and then preincubated for 0.5 h with Cytochalasin D (5 or 10 *μ*M), BHA (250 *μ*M), DPI (1 or 2 *μ*M), U0126 (30 *μ*M), SP600125 (50 *μ*M), or SN50 (50 *μ*M). The cells were then treated with 100 *μ*g/mL of a suspension of Asian dust particles, soil dust, or DMEM (negative control) for 6 h. TNF-*α* in the culture supernatants were assessed by means of an ELISA according to the manufacturer's instructions.

### 2.6. Evaluation of Reactive Oxygen Species (ROS) Production

RAW264.7 cells (1.5 × 10^4^ cells/well) were seeded in 96-well plates (Nunc), cultured at 37°C for 24 h, washed three times with phosphate buffered saline, and then incubated in phenol red-free DMEM containing 20 *μ*M 2′,7′-dichlorodihydrofluorescein diacetate (Cell Biolabs, Inc., San Diego, CA) for 30 min at 37°C. After incubation, the cells were treated with 100 *μ*g/mL a suspension of Asian dust particles, soil dust, or DMEM (for negative control) for 6 h and fluorescence was measured at 485 nm (subwave length, 530 nm).

### 2.7. Statistical Analysis

All results are expressed as mean ± SD. Differences were compared by using Bonferroni method.

## 3. Results and Discussion

### 3.1. Characteristics of the Asian Dust Particles and Soil Dust Samples

**Figure 1:**
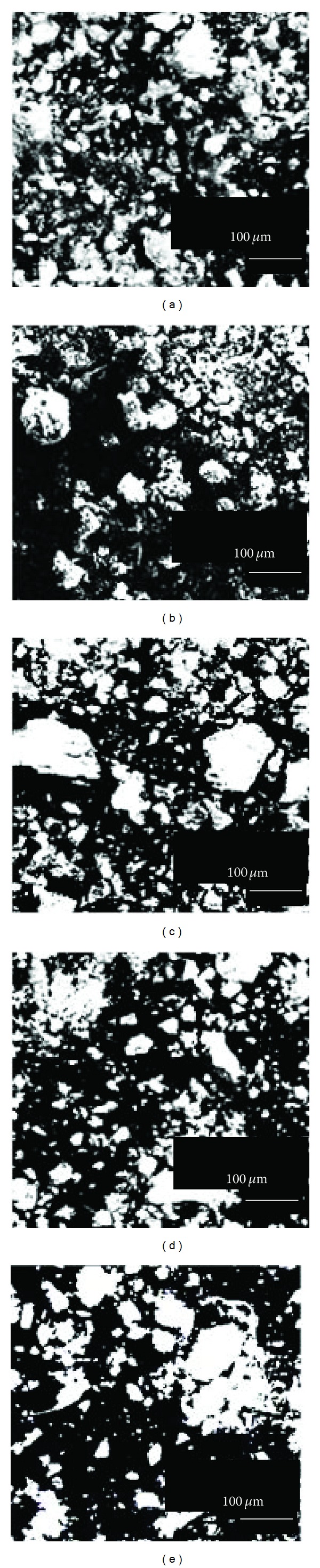
Scanning electron micrographs of two Asian dust particles (ADP1 (a) and ADP2 (b)) and three soil dust (SDP1 (c), SDP2 (d), and SDP3 (e)) samples. Prior to analysis, samples were adjusted to 0.25 mg/mL with deionized water and dried on a hotplate. Scale bars: 100 *μ*m.

First, the two Asian dust particles samples (ADP1 and ADP2) and three soil dust samples (SDP1, SDP2, and SDP3) were examined under a scanning electron microscope (Figure 1). We used soil dust samples as reference dust because satellite information indicated that the source regions of Asian dust are considered to be loess plateau, China [6]. Yamaguchi et al. demonstrated that all samples contained particles with a coarse surface texture, that the particles varied in form, and that some particles were condensed by scanning electron microscopic analysis. They also showed that silicon and aluminum were major components of Asian dust particles and that significant quantities of iron, calcium, and magnesium were also present [6]. In addition, large numbers of particles with a diameter ≥100 *μ*m were observed in the three soil dust samples (Figures 1(c), 1(d), and 1(e)) but were not observed in the two Asian dust particles samples (Figures 1(a) and 1(b)). Previously, Yamaguchi et al. showed that the mean particle size of Asian dust particles was <1 *μ*m [6]. It represented that the mean size of the Asian dust particles in our samples would be ≤1 *μ*m.

### 3.2. Asian Dust Particles Induce an Inflammatory Response in RAW264.7 Cells

Next, we evaluated the potential of Asian dust particles or soil dust to induce an inflammatory response in macrophages. Because our previous data show that particle-induced inflammatory responses are dependent on particle size [19–21], we also investigated whether the induction of an inflammatory response by soil dust was dependent on particle size by partitioning the particles with a diameter ≤10 *μ*m (SDP4) from SDP2.

**Figure 2:**
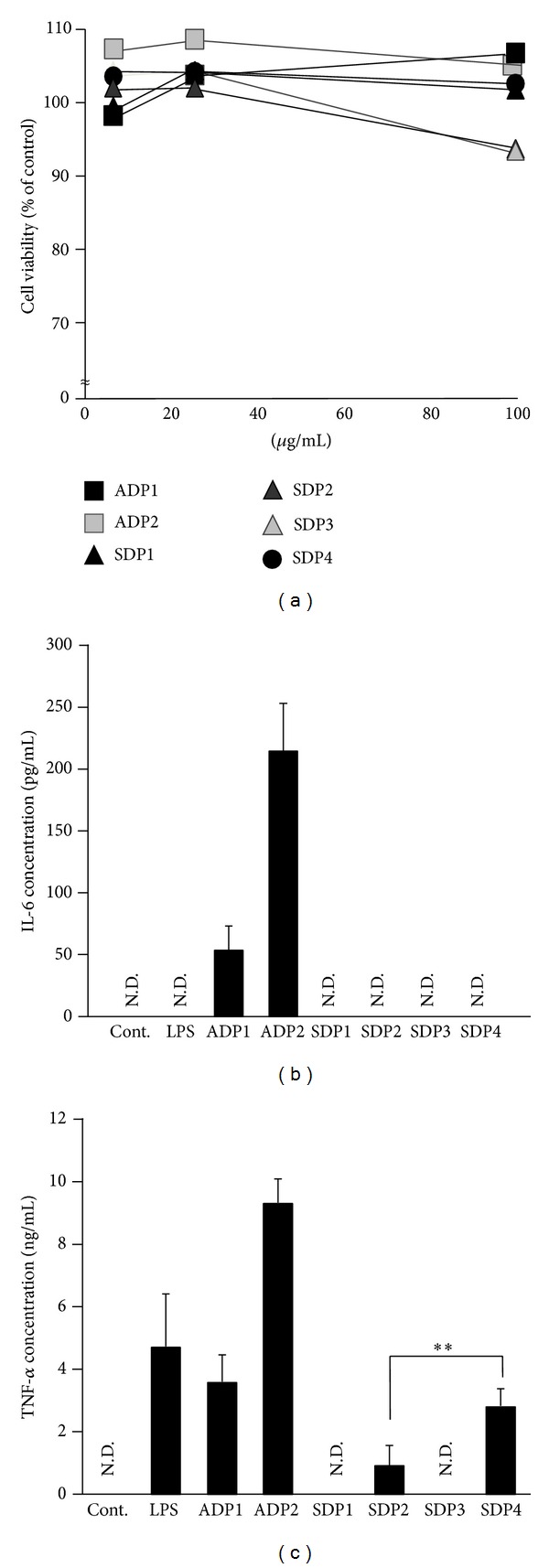
Assessment of cytotoxicity and inflammatory responses induced by Asian dust particles and soil dust in RAW264.7 cells. (a) RAW264.7 cells were treated for 24 h with 6.25, 25, or 100 *μ*g/mL of Asian dust particles ADP1, ADP2, or with soil dust SDP1, SDP2, SDP3, or SDP4 (particle diameter, ≤10 *μ*m). Cell viability was assessed by means of a methylene blue assay. (b, c) RAW264.7 cells were treated with 100 *μ*g/mL of each dust sample or 1.5 *μ*g/mL of lipopolysaccharide (LPS) for 24 h. Levels of interleukin-6 (IL-6) (b) and tumor necrosis factor-*α* (TNF-*α*) (c) in culture supernatants were assessed by means of enzyme-linked immunosorbent assay. DMEM was used as control (Cont.). Results are expressed as mean ± SD; *n* = 6; **P < 0.01; N.D., not detected.

To evaluate the cytotoxicity of the Asian dust particles and soil dust samples, RAW264.7 cells were treated with ADP1, ADP2 (Asian dust particles), SDP1, SDP2, SDP3 (soil dust), or SDP4 (soil dust; particle diameter ≤10 *μ*m) and cell viability was assessed. No significant cytotoxicity was observed in any of the experimental groups (Figure 2(a)).

Next, to examine the macrophage inflammatory response to Asian dust particles or soil dust, RAW264.7 cells were exposed to 100 *μ*g/mL of each sample for 24 h and cytokine production was assessed. The levels of IL-6 (Figure 2(b)) and TNF-*α* (Figure 2(c)) in the culture supernatants after treatment with ADP1 or ADP2 were markedly higher than those of the control group. Moreover, they were equivalent to or higher than those of lipopolysaccharide- (LPS-) treated group. However, the levels of IL-6 after treatment with SDP1, SDP2, or SDP3 were almost equal to those of the control group. In addition, the levels of TNF-*α* after treatment with SDP1 or SDP3 were almost equal to those of the control group but treatment with SDP2 could induce elevation of TNF-*α* production. Furthermore, the level of TNF-*α* in the culture supernatant after treatment with SDP4 was significantly higher than that after treatment with SDP2. These results suggest that Asian dust particles have greater potential to induce inflammation compared with soil dust and that particle size may affect the soil dust-induced macrophage inflammatory response. Materials such as lipopolysaccharides or *β*-glucan and chemicals such as ammonium or nitrates have been previously reported to adhere to Asian dust particles [5, 22]. Since the number of molecules that can adhere to particles per unit weight increases as particle size decreases, this may account for the increased production of IL-6 and TNF-*α* in macrophages exposed to smaller soil dust.

### 3.3. Asian Dust Particles-Induced ROS Production Mediates TNF-*α* Production in RAW264.7 Cells

**Figure 3:**
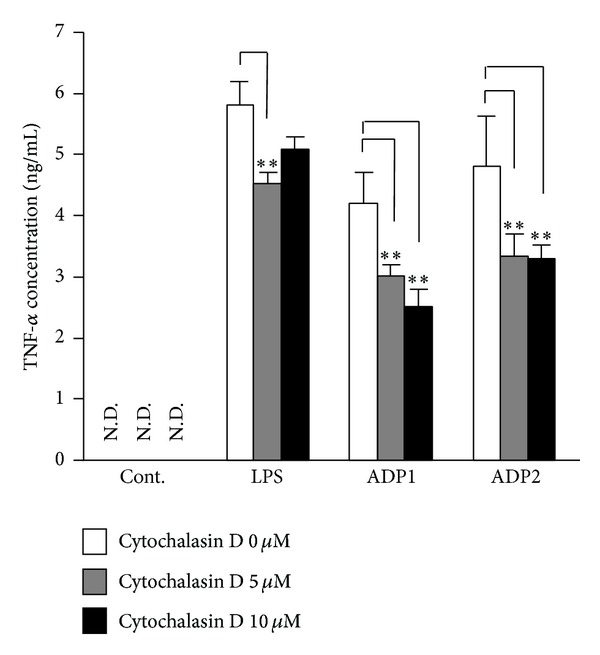
Cytochalasin D inhibited Asian dust particles-induced tumor necrosis factor-*α* (TNF-*α*) production in RAW264.7 cells. RAW264.7 cells were pretreated for 30 min with Cytochalasin D, an inhibitor of phagocytosis, and then treated for 6 h with 100 *μ*g/mL of Asian dust particles (ADP1 or ADP2) or 1.5 *μ*g/mL of LPS. Dimethyl sulfoxide (0.1%) vehicle was used as control (Cont.). The level of TNF-*α* in culture supernatants was assessed by means of an enzyme-linked immunosorbent assay. Results are expressed as mean ± SD; *n* = 6; **P < 0.01; N.D., not detected.

Next, to assess the mechanisms of the Asian dust particles-induced macrophage inflammatory response, we evaluated the association between inflammatory response and endocytosis. RAW264.7 cells were pretreated for 30 min with Cytochalasin D, an inhibitor of phagocytosis, and then treated for 6 h with 100 *μ*g/mL of ADP1 or ADP2; TNF-*α* production was assessed. Cytochalasin D significantly suppressed the production of TNF-*α* induced by ADP1 or ADP2 in a dose-dependent manner (Figure 3), suggesting that phagocytosis is a key aspect of the Asian dust particles-induced macrophage inflammatory response.

**Figure 4:**
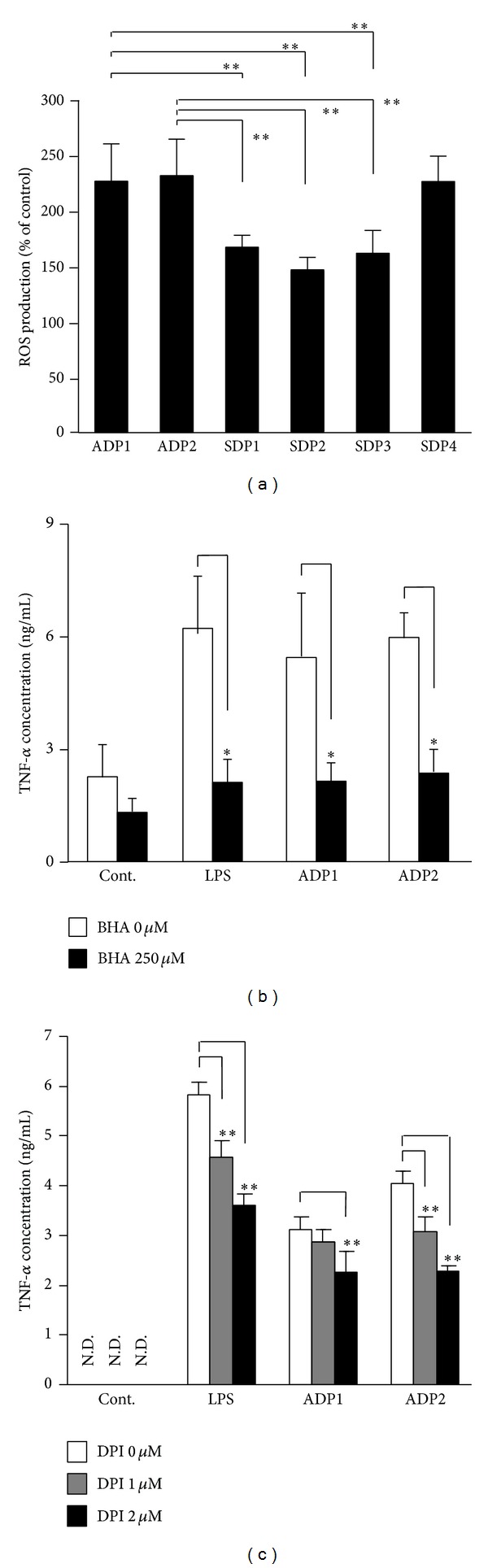
Asian dust particles-induced reactive oxygen species (ROS) production mediates tumor necrosis factor-*α* (TNF-*α*) production in RAW264.7 cells. (a) RAW264.7 cells were incubated in phenol red-free Dulbecco's modified Eagle's medium containing 20 *μ*M 2′,7′-dichlorodihydrofluorescein diacetate for 30 min. The cells were then treated for 6 h with 100 *μ*g/mL of one of the Asian dust particles (ADP1 or ADP2) or soil dust samples (SDP1, SDP2, SDP3, and SDP4) or culture medium. ROS production was measured as the fluorescence intensity of dichlorodihydrofluorescein. (b, c) RAW264.7 cells were preincubated for 30 min with (b) 250 *μ*M of butylated hydroxyanisole (BHA), a broad-spectrum ROS scavenger, or (c) 1 or 2 *μ*M of diphenyleneiodonium chloride (DPI), a specific inhibitor of NADPH oxidase. The cells were then treated for 6 h with 100 *μ*g/mL of ADP1 or ADP2 or 1.5 *μ*g/mL of LPS. Dimethyl sulfoxide (0.1%) vehicle was used as control (Cont.). The level of TNF-*α* in the culture supernatants was assessed by means of an enzyme-linked immunosorbent assay. Results are expressed as mean ± SD; *n* = 6; **P < 0.01; N.D., not detected.

ROS activate various signal pathways, such as the NF-*κ*B signaling pathway and the MAPK pathways, involved in the production of inflammatory cytokines [23, 24]. To evaluate the involvement of ROS in the macrophage inflammatory response to Asian dust particles, RAW264.7 cells were incubated with Asian dust particles (ADP1 or ADP2) or soil dust (SDP1, SDP2, SDP3, or SDP4) for 6 h and the fluorescence intensity of 2′,7′-dichlorodihydrofluorescein was measured as an index of ROS production. All dust samples induced ROS production (Figure 4(a)). ADP1 and ADP2 induced significantly higher ROS production compared with that induced by SDP1, SDP2, or SDP3. Furthermore, although not statistically significant, SDP4 did tend to induce greater ROS production than SDP1, SDP2, or SDP3, suggesting that particle size may be an important factor for soil dust-induced ROS production.

ROS are mainly produced by cell membrane, or endosome membrane, bound NADPH oxidase or by mitochondria [25, 26]; to investigate the involvement of ROS in Asian dust particles-induced TNF-*α* production, we measured TNF-*α* production induced by Asian dust particles in the presence of BHA, a broad-spectrum ROS scavenger, or DPI, a specific inhibitor of NADPH oxidase. BHA significantly suppressed the TNF-*α* production induced by ADP1 and ADP2 to almost the same level as that of the control group (Figure 4(b)). Furthermore, pretreatment with DPI also resulted in a dose-dependent decrease in the TNF-*α* production induced by ADP1 and ADP2 (Figure 4(c)). These results suggest that the Asian dust particles-induced production of inflammatory cytokines was mediated by ROS and that Asian dust particles-induced ROS production is dependent on NADPH oxidase.

### 3.4. Asian Dust Particles-Induced TNF-*α* Production Is Dependent on the MAPK and NF-*κ*B Signal Pathways

**Figure 5:**
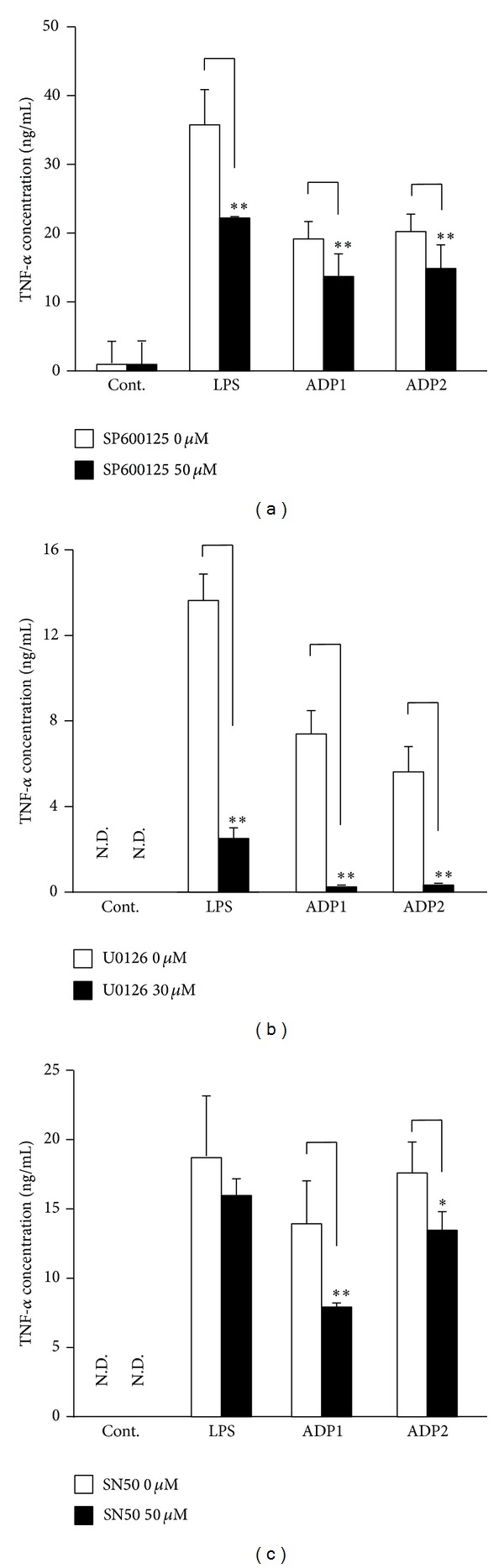
Asian dust particles-induced tumor necrosis factor-*α* (TNF-*α*) production is dependent on mitogen-activated protein kinase and nuclear factor-*κ*B signaling pathways. RAW264.7 cells were pretreated for 30 min with (a) 50 *μ*M of SP600125, an inhibitor of c-Jun N-terminal kinase; (b) 30 *μ*M of U0126, an extracellular signal-regulated kinase (ERK) inhibitor; or (c) 50 *μ*M of SN50, a nuclear factor-*κ*B (NF-*κ*B) inhibitor and then treated for 6 h with 100 *μ*g/mL of ADP1 or ADP2 or 1.5 *μ*g/mL of LPS. Dimethyl sulfoxide (0.1%) vehicle was used as control (Cont.). The level of TNF-*α* in the culture supernatants was assessed by means of an enzyme-linked immunosorbent assay. Results are expressed as mean ± SD; *n* = 6; *P < 0.05, **P < 0.01; N.D., not detected.

The MAPKs are a family of proteins that includes the p38, ERK, and JNK, which activate transcription factors involved in the production of inflammatory cytokines in response to external stresses or cytokine stimulation [27]. To investigate the association between the MAPKs and Asian dust particles-induced macrophage activation, RAW264.7 cells were incubated with ADP1 or ADP2 in the presence or absence of an inhibitor of the JNKs (SP600125) or ERKs (U0126), and the level of TNF-*α* in the cell culture supernatant was measured. Both SP600125 (Figure 5(a)) and U0126 (Figure 5(b)) significantly suppressed the TNF-*α* production induced by ADP1 or ADP2, suggesting that MAPK activation plays an important role in Asian dust particles-induced TNF-*α* production. Since the MAPKs stimulate transcription factors such as NF-*κ*B, which in turn regulate the expression of genes encoding cytokines such as IL-1*β*, IL-8, and TNF-*α*, RAW264.7 cells were incubated with ADP1 or ADP2 in the presence or absence of an NF-*κ*B inhibitor (SN50). SN50 significantly, but not completely, suppressed the TNF-*α* production induced by ADP1 or ADP2 (Figure 5(c)), indicating that NF-*κ*B may be partially involved in Asian dust particles-induced TNF-*α* production.

Asian dust particles contain chemical substances such as sulfates or nitrates derived from alkaline soil and microbiological materials [28] that may cause serious respiratory health problems in humans. Heat treatment of Asian dust particles has been reported to suppress allergic responses, which suggests that these adhered materials contribute to Asian dust particles-induced inflammation [29]. Our data suggests that as particle size decreases, the amount of chemicals or other materials that adheres to Asian dust particles increases, which may account for the different inflammatory responses to soil dust seen in the present study. Further data is needed on the attachment of these materials to Asian dust particles and on their effects on biological responses. Furthermore, it has been recently revealed that aerosols such as diesel exhaust or Asian dust particles contain not only microsized particles but also nanosized particles [30, 31]. Our recent research demonstrates that particles with diameter ≤100 nm show different biological responses and kinetics both* in vivo* and* in vitro* compared with microsized particles [32–34], suggesting that fine Asian dust particles have the potential to induce adverse biological effects. As shown in the present study, TNF-*α* production after treatment with SDP4 (soil sample; particle diameter, ≤10 *μ*m) was significantly higher than that after treatment with the other soil samples (SDP1, SDP2, or SDP3). This implies that particle size contributed to the macrophage inflammatory response to the soil samples; therefore, it is necessary to evaluate the biological effects of not only fine particles in the environment but also of fine particles with diameters ≤100 nm.

Recent studies have shown that crystalline silica disturbs the host immune system by activating the nucleotide-binding oligomerization domain, leucine-rich repeat pyrin domain containing 3 (NLRP3) inflammasome. The NLRP3 inflammasome has been examined for its role in the initial inflammatory response to a diverse range of stimuli [35–37]; however, the mechanisms of the activation of host immunity by Asian dust particles remain poorly understood. Therefore, further analyses are necessary to clarify this mechanism.

It has been reported that particulate matter with a diameter ≤1 *μ*m reaches the alveoli of the lung when aspirated [38]. However, there is little information on a global scale about the* in vivo* kinetics of these particles, including whether they infiltrate the body or not. The lack of data on particulate matter has raised concerns on their effect on human health. It is therefore important to clarify not only the* in vivo* kinetics and biological responses of tissues or cells to particulate matter with a diameter ≤1 *μ*m, but also the specific mechanisms of the biological effects of Asian dust particles.

## 4. Conclusions

Our results indicate that Asian dust particles have greater potential to induce inflammation compared with soil dust and that the size of soil dust particles affects soil sample-induced inflammatory responses. Furthermore, Asian dust particles-induced activation of macrophages is dependent on ROS production and involves the activation of the MAPK signal pathway.
